# MRI-based vertebral bone quality score for the assessment of osteoporosis in patients undergoing surgery for lumbar degenerative diseases

**DOI:** 10.1186/s13018-023-03746-0

**Published:** 2023-03-29

**Authors:** Zan Chen, Fei Lei, Fei Ye, Hao Yuan, Songke Li, Daxiong Feng

**Affiliations:** Department of Orthopaedics, The Affiliated Hospital of Southwest Medical University, No 25 TaiPing St, Jiangyang District, Luzhou, 646000 Sichuan People’s Republic of China

**Keywords:** Vertebral bone quality, Osteoporosis, Lumbar degenerative disease, DXA, Three-dimensional CT

## Abstract

**Purpose:**

To explore the value of vertebral bone quality (VBQ) scores in diagnosing osteoporosis in patients with lumbar degeneration.

**Methods:**

A retrospective analysis was conducted of 235 patients treated with lumbar fusion surgery at the age of ≥ 50; they were divided into a degenerative group and a control group according to the severity of degenerative changes on three-dimensional computed tomography. The L1-4 vertebral body and L3 cerebrospinal fluid signal intensities in the T1-weighted lumbar magnetic resonance imaging (MRI) image were recorded, and the VBQ score was calculated. Demographics, clinical data, and dual-energy X-ray absorptiometry (DXA) indicators were recorded, and the VBQ value was compared with bone density and T-score using the Pearson correlation coefficient. The VBQ threshold was obtained according to the control group and compared with the efficacy of osteoporosis diagnosis based on DXA.

**Results:**

A total of 235 patients were included in the study, and the age of the degenerative group was older than that of the control group (61.8 vs. 59.4, *P* = 0.026). The VBQ score of the control group suggested a higher correlation with the bone mineral density (BMD) value and T-score (*r* = − 0.611 and − 0.62, respectively). The BMD value and T-score in the degenerative group were higher than those in the control group (*P* < 0.05). Receiver-operating characteristic curve analysis showed that the VBQ score had a good predictive ability for osteoporosis (AUC = 0.818), with a sensitivity of 93% and a specificity of 65.4%. Among the undiagnosed osteoporosis patients with T-score, the VBQ score after adjusting the threshold was higher in the degenerative group (46.9% vs. 30.8%).

**Conclusions:**

Emerging VBQ scores can reduce the interference caused by degenerative changes compared to traditional DXA measures. Screening for osteoporosis in patients undergoing lumbar spine surgery provides new ideas.

## Introduction

Osteoporosis is a systemic bone disease characterized by low bone quality and degeneration of bone tissue microstructures [1, 2]. Patients with osteoporosis have a higher risk for lumbar degeneration, and in a previous study, approximately half of the women who underwent lumbar spine surgery had osteoporosis [3, 4]. The adverse consequences of loose internal fixation, adjacent vertebral fractures, and bone nonunion have improved our preoperative consideration of this type of patient [5–7].

Dual-energy X-ray absorptiometry (DXA) is recommended by the World Health Organization (WHO) for osteoporosis screening as a diagnostic tool [8]. DXA uses plane photography technology to scan absorption of the bone of the X-ray projection path, including the area bone density obtained from calcified blood vessels, the posterior structure of the spine, and spinal degeneration [9]. However, severe lumbar degenerative disease can lead to a false increase in the BMD value, masking the real situation of cancellous bone [10].

The vertebral bone quality (VBQ) score is a recently emerged magnetic resonance imaging (MRI)-based trabecular scoring method [11]. VBQ is used to measure fatty infiltration on noncontrast, T1-weighted images without the need for additional equipment and software, and it is an easy-to-use method. We delineated the designated area of interest of the L1-4 vertebral body and cerebrospinal fluid (CSF) and calculated the VBQ score. Theoretically, it is possible to avoid the interference of degenerative changes and reflect the true level of trabecular bone. Salzmann et al. studied patients undergoing lumbar spine surgery and found that VBQ had diagnostic value through quantitative computed tomography (QCT) [12]. However, QCT requires additional measurement software and investment, limiting its clinical implementation. There have been no studies reporting on the association of degressive changes causing fluctuations in DXA results with VBQ. In this study, patients were divided into degenerative and control groups, and the ability to diagnose osteoporosis with VBQ scores was evaluated.

## Materials and methods

### Patient cohort

This retrospective study was approved by the Ethics Committee of the Hospital. We reviewed 453 patients who underwent lumbar spine surgery for lumbar degenerative diseases from July 2019 to June 2020. Among them, lumbar scoliosis was defined as patients with coronal Cobb > 20°, obvious segmental instability, progressive neurological deterioration, and other surgical indications requiring surgical treatment. Inclusion criteria were as follows: (1) postmenopausal women and men older than 50 years; (2) lumbar fusion surgery for degenerative diseases of the lumbar spine; (3) DXA scan and lumbar spine three-dimensional computed tomography (CT) and MRI at the same time within 3 months before surgery; (4) < 2 vertebral fractures in the previous L1-L4. Exclusion criteria were as follows: (1) previous history of lumbar spine surgery and (2) history of metabolic bone diseases, ankylosing spondylitis, spinal infection, tumor, or radiation therapy.

### Variables

After screening out patients who met the criteria, demographic data were collected for each patient, such as age, sex, body mass index (BMI), comorbidities (diabetes, hypertension, rheumatic diseases), past smoking and drinking history, prior diagnosis of osteoporosis/osteopenia, and osteoporosis treatment (antiresorptive, anabolic therapy).

### Degenerative group condition

**Fig. 1:**
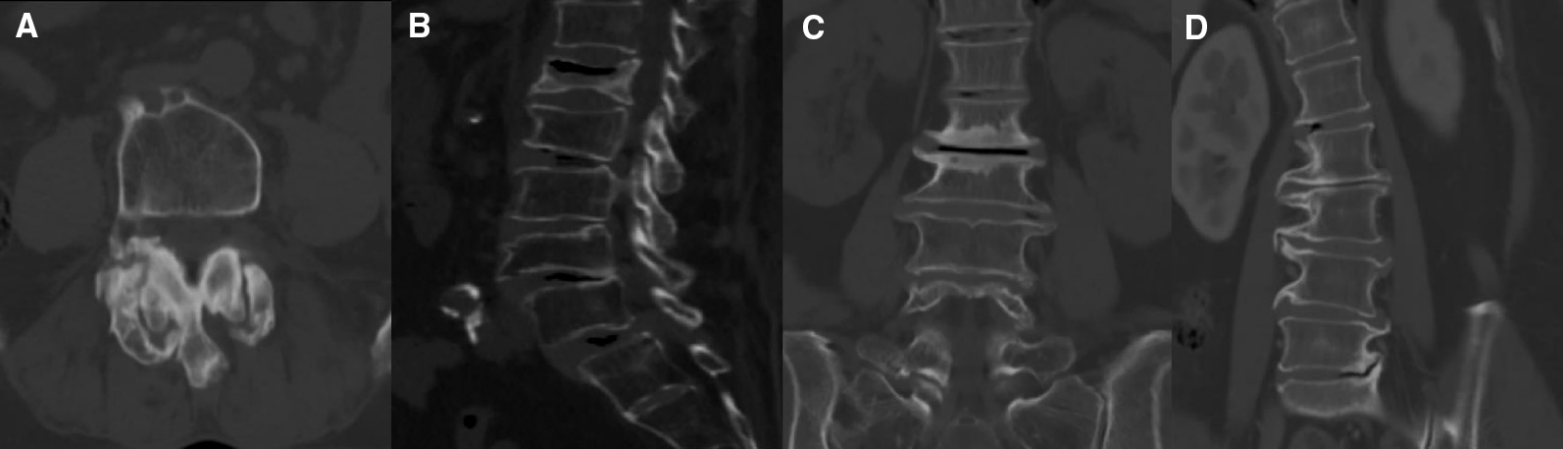
Typical degenerative changes: **A** severe hypertrophy of the articular process combined with subchondral cysts; **B** compression fractures; **C** end plate sclerosis; **D** claw osteophyte extending to adjacent disc > 2 mm

Patients receiving lumbar three-dimensional computed tomography (CT, Philips, 256-slice iCT machine, scan parameters: scan time 600 ms, matrix 512 × 512, slice spacing 0.625 mm, accuracy 0.1 mm) were screened for entry into the degenerative group, in reference to the L1-4 vertebral body. One of the following criteria and at least three degenerative vertebral bodies were required for inclusion in the degenerative group (Fig. 1):

1. Grade 2–3 for osteoarthritis of vertebral arthritis [13] (Table 1);
2. Ganet semiquantitative visual score 2–3 grade: vertebral body height reduction ≥ 25% [14];
3. UCLA Grading Scale for Intervertebral Space Degeneration III, IV: presence of intervertebral space stenosis with osteophytes, end plate sclerosis [15];
4. Extension direction of the osteophyte on the intervertebral disc space of > 2 mm Group C/D/E [16] (Table 1).

**Table 1:** Criteria for the inclusion of degenerative group

Grade	Criteria
*Grading osteoarthritis of the facet joints*	
0	Normal facet joint space (2 ± 4 mm width)
1	Narrowing of the facet joint space (< 2 mm) and/or small osteophytes and/or mild hypertrophy of the articular process
2	Narrowing of the facet joint space and/or moderate osteophytes and/or moderate hypertrophy of the articular process and/or mild subarticular bone erosions
3	Narrowing of the facet joint space and/or large osteophytes and/or severe hypertrophy of the articular process and/or severe subarticular bone erosions and/or subchondral cysts
*Direction of the formation of anterior lumbar vertebral osteophytes*	
A	No osteophytes
B	The pair of osteophytes extended in the direction of the adjacent disc
C	Almost complete bone bridge formation by a pair of osteophytes across the intervertebral disc space
D	The pair of osteophytes extended in a direction away from the adjacent disc
E	The osteophytes extended nearly horizontally to the vertebral body border without closing the intervertebral disc space
F	Ungroupable

The degree of degenerative disease in patients was determined independently by two trained researchers (C.Z. and F.L.). Intraobserver and interobserver reliability was analyzed.

### Bone density assessment

**Fig. 2:**
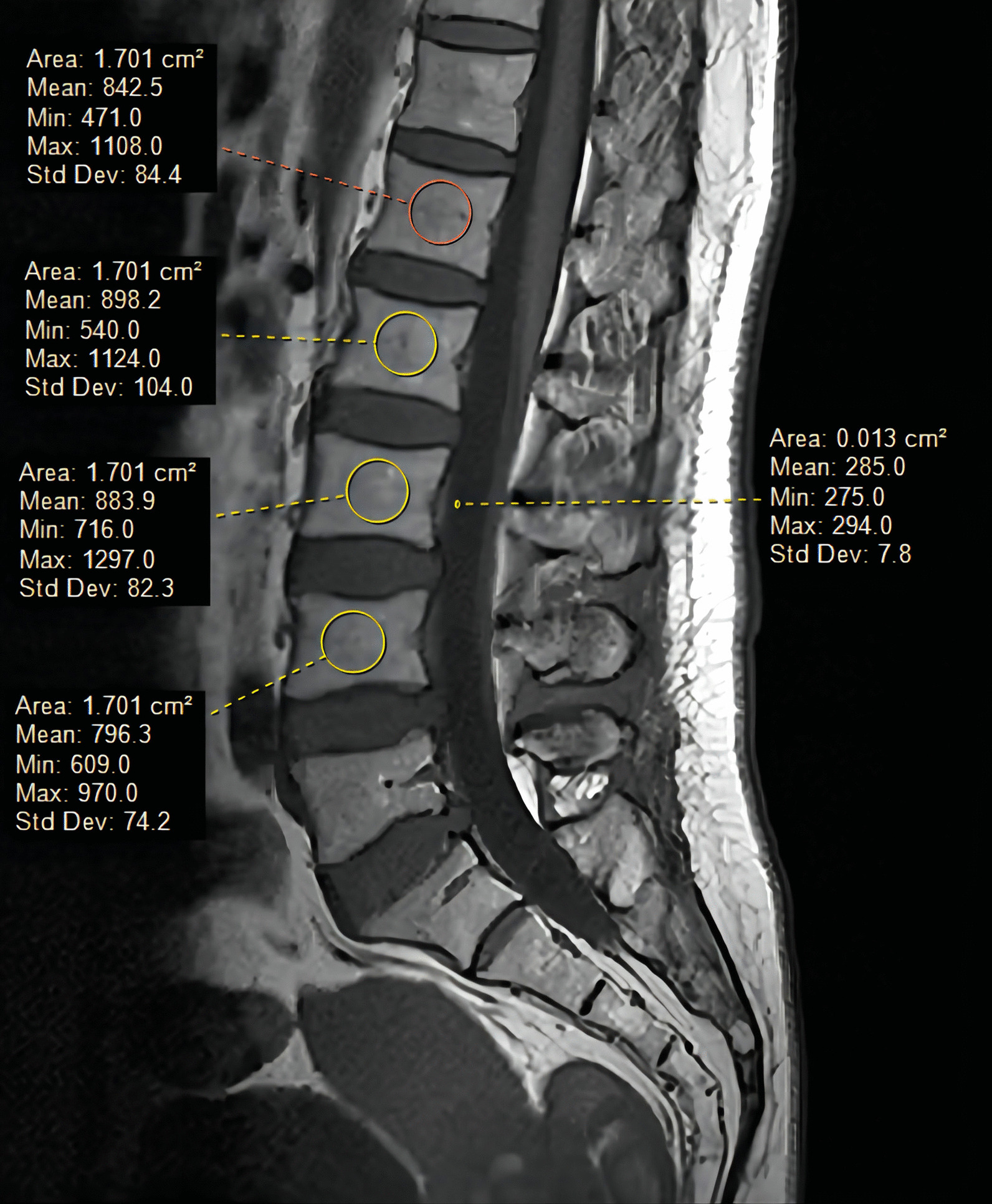
On sagittal non-T1-weighted images, a ring-shaped region of interest was placed in the corresponding region to display the SI to obtain the VBQ score

All patients were examined by DXA (GE, DPX Prodigy) of the lumbar spine (L1-4) and hip to obtain T-score and bone density (unit: g/cm^2^). The diagnostic criterion for osteoporosis is the lowest T-score of any measure of bone < − 2.5 [17]. The diagnosis of osteoporosis/osteopenia in patients with prior and current visits was recorded based on DXA criteria. In addition, 3.0 T MRI [Philips, Achieva, scanning parameters: T1WI (TR 600, TE 20), T2WI (TR 2 500, TE 100)] was carried out with the patient in the supine position. All images were transferred to the Image Archiving and Communication System (PACS) for viewing and analysis. VBQ measurements were taken using T1 noncontrast-weighted images [11]. Mid-sagittal slices are usually chosen, and parasural slices are chosen if scoliosis is present. A circular region of interest (ROI) was placed on the L1-L4 vertebral body (as shown in Fig. 2), avoiding the lesion and posterior venous plexus, and the mean signal intensity (SI) was recorded. If a collapsed vertebral body was present, the vertebral body level was excluded. The VBQ score was calculated using the remaining vertebral bodies. When measuring L3 CSF SI, if there was complete obstruction of the posterior CSF region, the adjacent segment CSF was chosen. First, the median L1-4 vertebral body SI value was calculated, and then the CSF SI was divided to obtain the relative VBQ value. The formula is as follows: VBQ score = SI_L1−L4_/SI_CSF_. To reduce measurement errors, the vertebral ROI was placed as small as possible in areas of the same size; the CSF ROI was also placed as small as possible. To verify the reliability of the VBQ score, 100 patients were randomly selected for evaluation. Two authors (C.Z. and H.Y.) performed the evaluation, and intraobserver and interobserver reliability was assessed. On the CT transverse scan, the ROI was placed on the middle layer of the L1 segment, including the trabecular bone as much as possible, avoiding cortical bone and heterogeneous areas such as venous plexuses and bone islets. The resulting measurements are presented in Hounsfield units (HU) [18].

### Modic changes

**Fig. 3:**
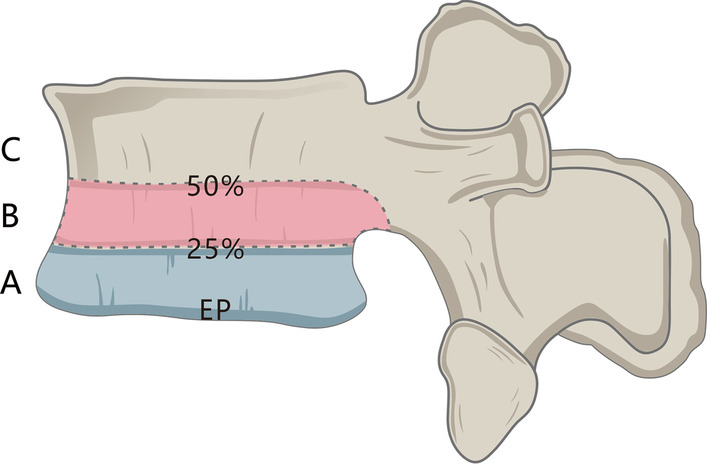
For grading according to the Modic changes in the height of the involved vertebral body on MRI, < 25% is grade A, between 25 and 50% is grade B, and > 50% is grade C

The VBQ score is relatively new, and the effect of Modic changes on the score has yet to be reported. For patients in our cohort, we recorded the Modic change type [19], segment number, and grade [20] in the L1-4 range on the MRI scan. We used a grading method that applies all Modic changes to grade the involved vertebral body height (see Fig. 3). A height of the affected vertebral body < 25% is grade A, between 25 and 50% is grade B, and > 50% is grade C.

### Statistical analysis

SPSS version 26 (SPSS, USA) was used for statistical analysis. If continuous variables/data conformed to the approximate normal distribution in terms of mean ± standard deviation (x ± s), the independent sample t test was used for comparison. Categorical data are expressed as percentages and were analyzed using the Chi-square test or Fisher’s exact probability test. The Pearson correlation coefficient was used to analyze the correlation between T-score, the BMD value, the HU value, and the VBQ score. Univariate analysis of covariance (ANCOVA) was used to compare differences between the two groups to control for the covariate age. A receiver operating characteristic curve (ROC) was used to analyze the differential value of the VBQ score in osteoporosis and calculate its specificity, sensitivity, negative predictive value (NPV), and positive predictive value (PPV). The Youden index was used to determine the cutoff value for VBQ to differentiate patients with osteoporosis and osteopenia. The correlation coefficient r is graded by an absolute number: 1 <| *r* |≤ 3 is defined as a weak correlation, 3 <| *r* |≤ 5 is a moderate correlation, and 5 <| *r* | is a strong correlation. The intraclass correlation coefficient (ICC) was employed to evaluate VBQ score intraobserver and interobserver reliability (ICC ≥ 0.8 defined as good reliability). Categorical variables were evaluated using kappa statistical tests. A *P* value < 0.05 was considered statistically significant.

## Results

A total of 235 patients treated with lumbar fusion surgery were ultimately included in the study. Among them, 101 were included in the degenerative group and 134 in the control group. Good intra- and interobserver reliability was observed according to the criteria for degenerative grouping (kappa = 0.819 and 0.809, respectively). There were 89 males and 146 females; the average age was 62.9 ± 8.9 years, and the oldest patient was 85 years. Among the different primary diagnoses, 143 (60.85%) had degenerative lumbar spinal stenosis, 47 (20%) had lumbar disc herniation, 35 (14.89%) had degenerative slippage, and 10 (4.26%) had degenerative scoliosis. Among all patients, the proportion of degenerative scoliosis included in the “degenerative group” was higher than that for other diagnoses (90% vs. 40.9%, *P* = 0.002). Two sets of detailed demographic and measured VBQ and DXA data were recorded (see Table 2).

**Table 2:** Characteristics of degenerative and control group data

	Degenerative group (*n* = 101)	Control group (*n* = 134)	*P* value	ANCOVA analysis of age-adjusted *P* value
Age(years)	61.8 ± 8.1	59.4 ± 7.8	0.026	–
Female	49 (48.5%)	97 (72.3%)	< 0.0001	–
BMI(kg/m2)	24.0 ± 3.2	23.9 ± 3.1	0.729	0.746
Smoking habits	4 (2.9%)	6 (5.9%)	0.433	–
Alcohol consumption	3 (2.2%)	2 (1.9%)	0.57	–
*Comorbidities*				
Diabetes	17 (12.6%)	13 (12.8%)	0.966	–
Hypertension	33 (24.6%)	28 (27.7%)	0.592	–
Rheumatic diseases	3 (2.2%)	4 (3.9%)	0.703	–
*Osteoporosis treatment*				
Antiresorptive medications	9 (8.9%)	11 (8.2%)	0.894	–
Anabolic medications	3 (3%)	5 (3.7%)	0.75	–
*Osteopenia/osteoporosis*				
Prior diagnosed	18 (17.8%)	22 (16.4%)	0.777	–
Current diagnosed	85 (63.4%)	66 (65.3%)	0.762	–
VBQ	2.9 ± 0.5	2.8 ± 0.5	0.012	0.047
HU Value	109.7 ± 37.6	130.2 ± 37.6	< 0.0001	0.001
L1-4 BMD(g/cm2)	1.015 ± 0.172	0.965 ± 0.151	0.018	0.006
L1-4 T-score	− 0.69 ± 1.50	− 1.15 ± 1.27	0.011	0.003
Femoral neck BMD(g/cm^2^)	0.848 ± 0.139	0.86 ± 0.119	0.478	0.98
Femoral neck T-score	− 0.85 ± 0.14	− 0.64 ± 1.01	0.157	0.484
Total hip BMD(g/cm^2^)	0.908 ± 0.146	0.907 ± 0.120	0.984	0.625
Total hip T-score	− 0.57 ± 1.09	− 0.53 ± 0.92	0.755	0.865
Lowest BMD(g/cm^2^)	0.821 ± 0.136	0.833 ± 0.115	0.444	0.888
Lowest T-score	− 1.41 ± 1.12	− 1.48 ± 1.04	0.605	0.284

Among the two groups, the degenerative group was older (61.8 vs. 59.4, *P* = 0.026), and the control group was more likely to be female (72.3% vs. 48.5%, *P* < 0.0001). There were no statistically significant differences in other demographic characteristics, comorbidities, or anti-osteoporotic drug use. We noted that HU values in the degenerative group were lower than those in the control group (109.7 vs. 130.2, *P* < 0.0001), but that the degenerative group had a higher VBQ score (2.9 vs. 2.8, *P* < 0.05). After age adjustment, the two groups showed significant differences in VBQ score, HU value, L1-4 BMD value, and T-score. The HU value correlated inversely with the VBQ score, with a Pearson correlation coefficient > − 0.6 (Table 3). In addition, L1-4 BMD values and T-scores in the degenerative group were higher than those in the control group (1.015 vs. 0.965, − 0.69 vs. − 1.15, respectively).

**Table 3:** Correlation between VBQ score and other bone density in two groups (r value)

	Degenerative group	Control group
L1-4 BMD	− 0.453*	− 0.611*
L1-4 T-score	− 0.460*	− 0.620*
L1 HU value	− 0.630*	− 0.611*

*r value corresponding to p value < 0.05

**Fig. 4:**
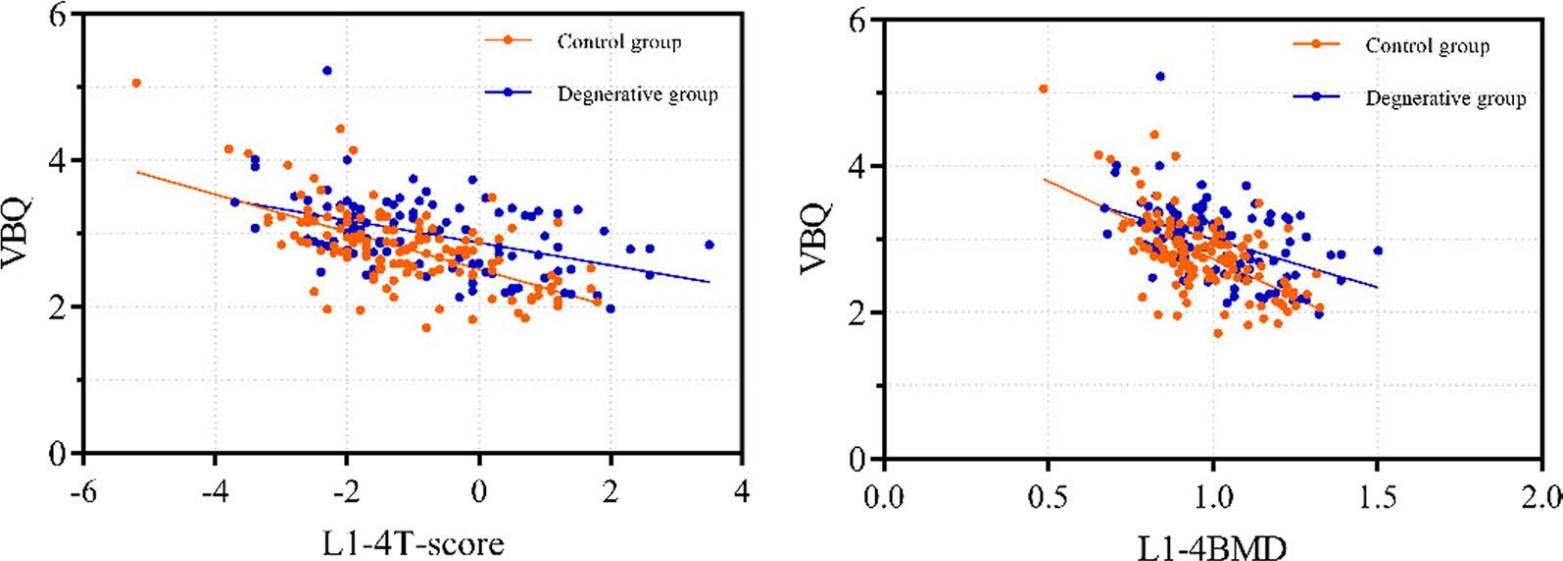
VBQ scores correlated with the L1-4 T score and BMD values in the two groups

**Fig. 5:**
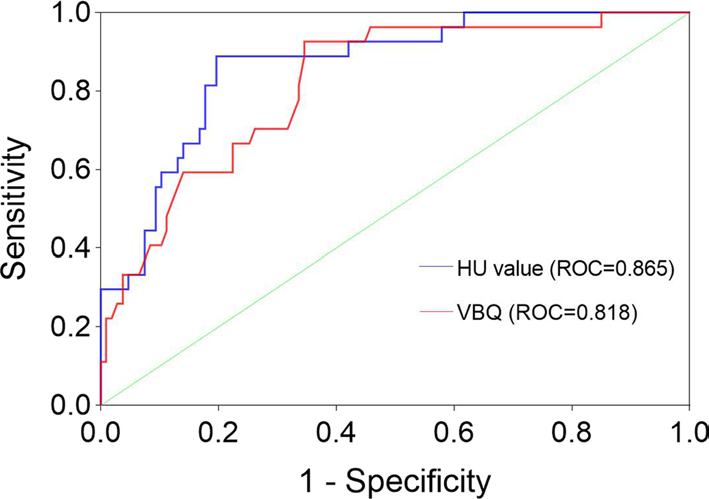
In the control group, the two noninvasive methods suggested different ROC curves. As a diagnostic method for osteoporosis, the sensitivity of the HU value was 80.4%, and the specificity was 88.9%. The sensitivity of the VBQ score was 93%, and the specificity was 65.4%

Two groups of VBQ scores correlated inversely with DXA measurements of BMD values or T-scores (p < 0.05), with the control group, suggesting a higher correlation with either (Table 3). The relationship between the two sets of VBQ scores and T-scores and BMD values was visualized by a scatter plot (Fig. 4). For the overall correlation (all *P* < 0.0001), the VBQ score and the femoral neck and hip T-score showed a moderate correlation (*r* = − 0.372 and − 0.438, respectively); the overall L1-4 and lowest T-score showed a high correlation (*r* =− 0.501 and − 0.537, respectively). Using ROC to analyze HU value and VBQ score  (Fig. 5), the area under the curve (AUC) as a diagnostic tool for osteoporosis was 0.865 (95% confidence interval, 0.794–0.9037) and 0.818 (95% confidence interval, 0.734–0.902), respectively. The VBQ score for osteoporosis/osteopenia was 86.8% for PPV and 50% for NPV. Based on the control group data, the corresponding thresholds for osteopenia (− 1 < T < − 2.5) and osteoporosis (T < − 2.5) were calculated and adjusted to one decimal place for the threshold (Table 4).

**Table 4:** In the control group, the VBQ score was diagnostic of osteoporosis and osteopenia

Criterion	VBQ threshold	Adjustment threshold	Sensitivity (%)	Specifcity(%)	AUC (95% CI)
Osteopenia	2.56	2.6	83.50	53.10	0.733 (0.645–0.821)
Osteoporosis	2.83	2.9	93	65.40	0.818 (0.734–0.902)

According to the adjusted threshold criteria of the VBQ score, osteoporosis was higher in the degenerative group among patients undiagnosed with osteoporosis by T-score (46.9% vs. 30.8%). When T-score was used as the standard, there was no significant difference in prevalence between the two groups (19.8% vs. 20.1%, *P* = 0.541); when the VBQ score was used as the standard, the prevalence in the degenerative group was significantly higher than that in the control group (53.5% vs. 38.8%, *P* = 0.025). When VBQ criteria were used to explore diagnosis of different diseases, there was no significant difference between diagnoses (*P* = 0.121). Notably, degenerative scoliosis showed an osteoporosis rate of 80% (8/10). VBQ scores had good intraobserver and interobserver reliability, with ICCs of 0.846 and 0.835, respectively.

A total of 101 (42.9%) patients exhibited Modic changes, of which type 2 was the most common, in 76.2% (77/101), and the most common occurred in a single segment, in 43.6% (44/101). In grading the degree of impact of Modic changes, the largest number occurred at grade A (72/101). When comparing the two groups, there were no statistically significant differences in the type of Modic change, the involved segment, and the involved grading (Table 5).

**Table 5:** Characteristics of Modic changes in control and degeneration groups

	Degenerative group (*n* = 56)	Control group (*n* = 45)	*P* value
*Modic type*			
Type 1	4 (7.1%)	2 (4.4%)	
Type 2	39 (69.6%)	38 (84.4%)	
Type 3	4 (7.1%)	4 (8.9%)	0.099
Mixed type	9 (16.1%)	1 (2.2%)	
*Number of affected segments*			
1 level	16 (28.6%)	28 (62.2%)	
2 levels	21 (37.5%)	10 (22.2%)	
3 levels	15 (26.8%)	6 (13.3%)	0.007
4 levels	4 (7.1%)	1 (2.2%)	
*Modic grading*			
Grade A	40 (71.4%)	32 (71.1%)	
Grade B	8 (14.3%)	11 (24.4%)	0.149
Grade C	8 (14.3%)	2 (4.4%)	

## Discussion

The patients in this study were divided into a degenerative group and a control group according to the severity of degeneration, and the difference in the VBQ score and T-score between the two groups was explored. Osteoporosis and degenerative changes are two factors that increase risk with age [21], but their combination can produce contradictory bone evaluation results. Measurements of ROIs in specific myeloid regions based on VBQ scores have the advantage of undisturbed degenerative changes over traditional DXA techniques. According to DXA, the degenerative group had higher lumbar BMD values, which correlated poorly with the VBQ score. Conversely, the higher correlation between VBQ scores and bone mineral density in the control group may indicate that the VBQ score more realistically reflects bone quality status. This is the first report on the diagnostic power of VBQ scores for degenerative effects in patients undergoing lumbar surgery.

Central DXA is the most commonly used bone density measurement, but it has technical limitations and subject interference [17, 22]. DXA overestimation of degenerative changes may result in missed diagnoses, with 43.5% (44/101) of the degenerative group identified based on the VBQ threshold in this study, and 66.6% (4/6) of patients with lumbar scoliosis had missed diagnoses. The grouping of degenerative changes is based on the report of Muraki et al. [10] who reported that factors affecting the increase in bone density of the lumbar spine, such as osteophytes, bone sclerosis, and intervertebral disc stenosis, can be found on X-ray. Our research builds on this.

In past reports, less than half of screening rates for patients with potential suspicion of osteoporosis among spinal surgeons have been reported, and 74% of doctors who have access to bone density data will change the options for surgery and treatment [23]. In another report, Chin et al. [24] analyzed 68% of 759 patients over 50 years of age who underwent surgery. Low screening rates for men may be related to the age recommended by guidelines. As a routine noninvasive examination before lumbar spine surgery, lumbar spine MRI does not require additional equipment, radiation exposure, etc., and provides a possible solution for assessing bone quality.

Bone quality loss occurs earlier in trabecular bone, which may cause changes in fracture risk and axial mechanics and is of great concern in osteoporosis monitoring [2, 25]. Adipocytes replaced in osteoporotic bone show a high signal in trabecular bone on T1-weighted images, which provides a theoretical basis for MRI to evaluate bone quality [26, 27]. Correlations between different MRI measurements and bone mineral density have also been demonstrated in other studies [28, 29]. On this basis, Ehresman et al. overcame the difference in the baseline signal of the MR system and obtained the VBQ score by calculating the intrinsic difference in CSF signal adjustment, which was applied to bone quality evaluation [11]. At the same time, interrater and intrarater evaluations of trainers at different stages have good reliability [30]. Good reliability was also observed in our study. VBQ scores have been used to predict fragility fractures and new fractures with spinal metastases in patients [31, 32]. In another recent study, bone density represented only one dimension of bone strength, with microcomputed tomography (μCT) used as the gold standard to assess three-dimensional bone morphology [33, 34]. The VBQ score suggests a correlation between qualitative and quantitative dimensions of bone microstructures, and it may provide additional bone quality characteristics. VBQ scores have value and advantages in a variety of scenarios, but no studies have shown the ability and value of VBQ scores to diagnose bone quality with degenerative changes in the lumbar spine, which is very different for the two evaluation methods; both bone quality evaluations showed good predictive ability to avoid degenerative regions. Another recognized noninvasive measure of CT (HU) was included in our study [18, 35]. There are advantages over traditional DXA. Although both can be prospectively used by radiologists, MRI measurement training and time costs are higher.

The purpose of this article is to discuss the diagnostic value of VBQ scores in patients with osteoporosis. In terms of AUC, both the VBQ score and HU value have good predictive ability (AUC = 0.818 and 0.865, respectively), with that of the latter being slightly stronger. However, the sensitivity of the VBQ score was higher. For VBQ scores, the high sensitivity of the resulting threshold is appropriate for high-risk populations with decreased bone quality and poor ability to identify negative events. Low specificity may include cases of excessive osteopenia (73.6%). In the degenerative group, more than 1 in 3 (37.6%) patients were diagnosed with osteoporosis, 50% had osteopenia, and early, difficult-to-identify osteopenia was common. More false-negative outcomes in patients with degenerative scoliosis also reflect a higher degree of degenerativeness, increasing the emphasis on degenerative changes. In addition, patients older than 60 years had higher screening rates (51.5% vs. 34.5%) among patients not included in T-score osteoporosis. For advanced age with bone density mismatch being common, the VBQ score provides a good complement. In general, significant bone quality loss may occur in untreated patients. Regardless of baseline bone density status, significant bone loss increases the risk of fracture and may require early intervention [36–41].

Haffer et al. enrolled 180 patients with a VBQ score of 2.57 in a healthy group and 3.04 in an osteoporosis/osteopenia group by reference to QCT [34]. However, they argue that with reference to more accurate QCT measurements, the resulting bone quality score is lower than other studies predicted and that QCT may not be a widely applicable reference standard. In another study, only femoral neck and total hip T-scores were selected as reference criteria [12, 31]. More emphasis was placed on lumbar bone density in the present study, suggesting a higher correlation with VBQ scores than in other studies, which indicates the reliability of bone quality scores. Our analysis is based on the most commonly used DXA as a reference standard, ruling out the limitations of degenerative changes. The VBQ threshold for osteoporosis was different from other studies, and such differences may be due to several factors, such as race, bone density reference standard, and scanner types.

At the same time, we compared normal with osteoporosis/osteopenia and found that the VBQ score and the T score suggested a higher correlation for the osteoporosis group. This also indirectly illustrates that T1-weighted images reflect the degree of fat infiltration of sparse bone trabecula, though the high correlation in the control group may indicate a synergistic effect of this bone quality loss. Interestingly, Li et al. conducted age stratification comparisons in a study of patients with osteoporosis compression fractures [42]. DXA results rather than VBQ scores suggested differences between age groups, indicating spatiotemporal differences in bone mineral loss and fatty infiltration. However, in another study of patients with lumbar hardware failure and adjacent vertebral degeneration, VBQ scores rather than DXA outcomes were used as predictors of resurgery [43]. The results of the two bone quality evaluations in the current study are inconsistent, and the mechanisms for bone remodeling and trabecular loss are very complex. We do not recommend alternatives to these techniques, and different tools are necessary for interpretation and supplementation.

This article reports Modic changes, which are changes in the bone marrow of the subchondral vertebrae [20]. Bone marrow edema/changes caused by Modic changes can interfere with bone quality assessment to some extent, especially for vertebral bodies with grade 2/3. If there is a high-grade Modic change in a single segment, in the VBQ scoring criterion, the median result of the vertebrae may be used to reduce the interference of this type of vertebral body SI. However, for cases of long-segmental high-grade Modic changes, VBQ scoring may not be a suitable option. In our study, grade A accounted for the highest proportion, but the class A-affected range was small, with most appearing at the edge, which was not affected when delineating the circular ROI. The impact of high-grade Modic changes on VBQ and whether Modic areas can be avoided need to be further explored.

VBQ measurement is limited, measuring the potential mean of all vertebral body levels in osteoporosis patients and excluding the lowest values. However, diagnosis of DXA recommends including the minimum value to avoid mismatches between different sites. MRI is based on sagittal measurements and requires exclusion of fracture levels, with multiple lumbar vertebral fractures leading to measurement termination. There are also limitations in this study. First, factors such as abdominal vascular calcification and bone islands were not included in degenerative changes. Second, sex bias may be present, with a higher proportion of women in the control group, which may interfere with the final results. We also obtained VBQ scores with low specificity, and higher specificity thresholds are needed for inclusion and increased applicability for different populations in the future.

## Conclusion

The VBQ score enhances screening for osteoporosis. For findings of significantly increased osteoporosis/osteopenia over 60 years of age, degenerative changes in coverage, in combination with DXA, act as a supplement to bone quality testing.

## Data Availability

The datasets used and/or analyzed during the current study are available from the corresponding author on reasonable request.

## Abbreviations

VBQ: Vertebral bone quality
CT: Computed tomography
CSF: Cerebrospinal fluid
MRI: Magnetic resonance imaging
DXA: Dual-energy X-ray absorptiometry
BMD: Bone mineral density
QCT: Quantitative computed tomography
HU: Hounsfield unit
ROI: Region of interest
SI: Signal intensity
ROC: Receiver operating characteristic curve
AUC: Area under curve
NPV: Negative predictive value
PPV: Positive predictive value
ICC: Intraclass correlation coefficient
μCT: Microcomputed tomography
